# Nonalcoholic Fatty Liver Disease-Related Hepatocellular Carcinoma: Clinical Patterns, Outcomes, and Prognostic Factors for Overall Survival—A Retrospective Analysis of a Slovak Cohort

**DOI:** 10.3390/jcm10143186

**Published:** 2021-07-20

**Authors:** Dominik Safcak, Sylvia Drazilova, Jakub Gazda, Igor Andrasina, Svetlana Adamcova-Selcanova, Radovan Barila, Michal Mego, Marek Rac, Lubomir Skladany, Miroslav Zigrai, Martin Janicko, Peter Jarcuska

**Affiliations:** 1 Department of Radiotherapy and Oncology, East Slovakia Institute of Oncology, Rastislavova 43, 041 91 Kosice, Slovakia; safcak@vou.sk (D.S.); andrasina@vou.sk (I.A.); 2 Internal Medicine Department, Hospital Poprad a.s., Banicka 803, 058 01 Poprad, Slovakia; 3 2nd Department of Internal Medicine, P. J. Safarik University and L. Pasteur University Hospital, Trieda SNP 1, 040 11 Kosice, Slovakia; jakub.gazda@upjs.sk (J.G.); martin.janicko@upjs.sk (M.J.); peter.jarcuska@upjs.sk (P.J.); 4 2nd Department of Internal Medicine, HEGITO, F. D. Roosevelt University Hospital, Namestie L Svobodu 1, 975 17 Banska Bystrica, Slovakia; sselcanova@gmail.com (S.A.-S.); lubomir.skladany@gmail.com (L.S.); 5 Oncological Cluster, Stefan Kukura Hospital in Michalovce, Spitalska Ulica 2, 071 01 Michalovce, Slovakia; radovan.barila@gmail.com; 6 2nd Department of Oncology, Faculty of Medicine, Comenius University and National Oncology Institute of Slovakia, Klenova 1, 833 10 Bratislava, Slovakia; misomego@gmail.com; 7 Department of Internal Medicine, Teaching Hospital Nitra, Spitalska 6, 949 01 Nitra, Slovakia; marek.rac@fnnitra.sk; 8 1st Department of Internal Medicine, Ladislav Derer University Hospital in Bratislava, Limbova 5, 833 05 Bratislava-Kramare, Slovakia; miroslav.zigrai@szu.sk

**Keywords:** nonalcoholic fatty liver disease, nonalcoholic steatohepatitis, cirrhosis, hepatocellular cancer, overall survival, prognostic factors

## Abstract

Objective: To compare NAFLD-related HCC and other etiology-related HCC and to describe predictive factors for survival in patients with NAFLD-related HCC independent of the BCLC staging system. Methods: We performed a multicenter longitudinal retrospective observational study of patients diagnosed with HCC during the period from 2010 through 2016. Results: 12.59% of patients had NAFLD-related HCC, and 21.91% had either NAFLD or cryptogenic etiology. NAFLD-related HCC patients were younger (*p* = 0.0007), with a higher proportion of women (*p* < 0.001) compared to other etiology-related HCC patients. The NAFLD group had a significantly lower proportion of patients with liver cirrhosis at the time of HCC diagnosis (*p* < 0.0001), and they were more frequently diagnosed with both diabetes and metabolic syndrome when compared to other etiology-related HCC (*p* < 0.0001). We did not find any difference in the overall survival or in the progression-free survival between NAFLD-related and other etiology-related HCC patients staged as BCLC B and BCLC C. NAFLD-related HCC patients with three or more liver lesions had a shorter overall survival when compared to patients with one or two liver lesions (*p* = 0.0097), while patients with baseline CRP values of ≥5 mg/L or with PLR ≥ 150 had worse overall survival (*p* = 0.012 and *p* = 0.0028, respectively). ALBI Grade 3 predicted worse overall survival compared to ALBI Grade 1 or 2 (*p* = 0.00021). In NAFLD-related HCC patients, PLR and ALBI remained significant predictors of overall survival even after adjusting for BCLC. Conclusion: NAFLD-related HCC patients have a similar prognosis when compared to other etiology-related HCC. In NAFLD-related HCC patients, ALBI and PLR are significant predictors of the overall survival independent of the BCLC staging system.

## 1. Introduction

Primary liver tumors are the sixth most common malignancy worldwide and the third leading cause of cancer-related death [1]. The most common primary liver tumor is hepatocellular carcinoma (HCC). HCC arises in the vast majority of patients in the setting of liver cirrhosis, but it can also arise in patients without liver cirrhosis. The most common underlying diseases that lead to HCC are alcoholic liver disease, chronic hepatitis B, and chronic hepatitis C. Recently, however, the number of patients with HCC in nonalcoholic fatty liver disease (NAFLD) has been increasing. The presence of metabolic syndrome and type 2 diabetes mellitus may also increase the risk of HCC in liver diseases of other etiologies [2]. NAFLD is defined as excessive fat accumulation in the liver and the presence of steatosis in >5% of hepatocytes, while secondary causes of hepatic steatosis, including alcohol consumption, are excluded [2]. The prevalence of NAFLD in Europe is approximately 24%, with variations from 5% to 44% across countries [3]. NAFLD can progress to nonalcoholic steatohepatitis (NASH) with the presence of inflammation and ballooning, with or without fibrosis. NASH progresses to liver cirrhosis in a significant proportion of patients [4]. Cirrhosis of the liver can develop in 15–30% of patients with NAFLD, and patients with NAFLD cirrhosis are usually older than those with cirrhosis of other etiologies [5]. HCC can arise in a cirrhotic liver, but can also arise in NASH without cirrhosis, or even in simple steatosis. Approximately 15–50% of HCCs arise in NAFLD patients in a non-cirrhotic liver [4]. The annual incidence of NAFLD-related HCC in Europe and the USA is 0.21–0.6/1000 patients [6,7,8]. In Europe, the annual incidence of NASH-related HCC is 1.11–1.32 [7]. The incidence of HCC depends on the stage of NAFLD. The annual incidence of all patients with NAFLD in a North American study was 0.21/1000 patients, with 0.08/1000 patients without cirrhosis and up to 10.6/1000 patients with cirrhosis [8]. The pathophysiology of NAFLD-related HCC is multifactorial. Genetic and epigenetic factors, including age, gender, insulin resistance, subclinical inflammation and fibrosis in the liver parenchyma, immune system status, intestinal microbiota, and lifestyle and environmental factors play a role in the development of HCC [4,5,9,10,11,12]. The aim of our study was to compare NAFLD-related HCC and HCC in other liver diseases, as well as to describe independent predictive factors for survival in patients with NAFLD-related HCC.

## 2. Materials and Methods

We performed a multicenter longitudinal retrospective observational study of patients diagnosed with HCC at eight specialized centers in Slovakia during the period from 2010 through 2016 (Banska Bystrica, Bratislava (2), Kosice (2), Michalovce, Nitra, and Poprad). The inclusion criterion was the diagnosis of HCC consistent with the EASL-EORTC guidelines (HCC confirmed by either histopathological examination or magnetic resonance imaging) [13]. The exclusion criteria were as follows: (a) Etiology not available, (b) cryptogenic etiology, and (c) combined or rare etiologies. 

NAFLD was diagnosed by abdominal ultrasound, computer tomography scans, or magnetic resonance imaging. All NAFLD patients were both HBsAg- and anti-HCV- negative, and alcohol consumption in this group was less than 20 g per day in women and less than 30 g per day in men. We only included those NAFLD-related HCC patients who had already been diagnosed with NAFLD before the diagnosis of HCC.

**Figure 1:**
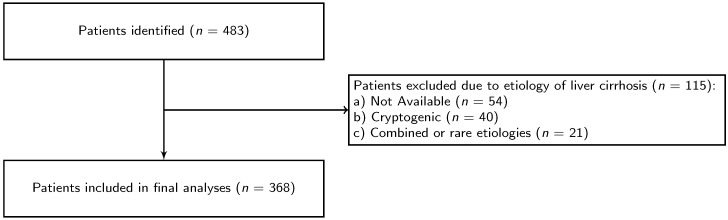
Flowchart of patient inclusion.

Initially, we screened all patients diagnosed with a malignant neoplasm of the liver and intrahepatic bile ducts (ICD-10-CM C22) and identified 483 patients with a diagnosis of liver cell carcinoma (ICD-10-CM C22.0). Fifty-four patients did not have any available information on the etiology; on the contrary, the etiology was well documented in 429 patients. Of these patients, 54 patients had NAFLD-related HCC (12.59%) and 94 patients (21.91%) had either NAFLD or cryptogenic etiology. Altogether, out of all 483 identified patients, 116 patients met one of the exclusion criteria; therefore, 368 patients were included in the final analysis (Figure 1).

Case report forms (CRFs) were completed by D.S. with the on-call assistance of P.J. Apart from the abovementioned information, the CRFs included baseline blood test results, which were also later used to calculate the neutrophil-to-lymphocyte ratio (NLR) [14], the platelet-to-lymphocyte ratio (PLR) [15], the serum aspartate aminotransferase-to-platelet ratio index (APRI) [16], and the albumin–bilirubin grade (ALBI) [17]. If any condition that may have influenced baseline values were present (e.g., acute bacterial infection or corticosteroid treatment), repeated analyses were performed after the restoration of that condition (including the wash-out period in case of medication) and new results were extracted.

For this study, we also obtained the patients’ history of diabetes and/or metabolic syndrome and patients’ exposure to low molecular weight heparin (both prophylactic and therapeutic indications), metformin, statins, and ursodeoxycholic acid. Diabetes was defined according to American Diabetes Association, and metabolic syndrome was defined according to Alberti et al. [18,19].

Patients were diagnosed with liver cirrhosis using ultrasonography, magnetic resonance imaging (MR), or histopathological examination of the resected/explanted liver parenchyma. The Child-Pugh score was calculated to estimate cirrhosis severity, and performance status was evaluated using the Eastern Cooperative Oncology Group Scale [20]. CT scans of the thorax, abdomen, and pelvis were used to identify potential extrahepatic spread. All centers used the Barcelona Clinic Liver Cancer (BCLC) staging system to guide the management of patients [21]. 

Treatment response was evaluated using CT scans or MR imaging according to the modified Response Evaluation Criteria in Solid Tumor [22]. Progression-free survival was defined as the time from inclusion until objective tumor progression or death, whichever occurred first. Disease-free survival was defined as the time from inclusion until disease recurrence or death from any cause [23]. The CRFs also included the date of death extracted either from the patients’ medical records or from the database of the Slovak Health Care Surveillance Authority. Finally, we followed patients up until they died. Thus, the duration of the follow-up equals the time of the overall survival.

The study protocol was in accordance with the 1964 Declaration of Helsinki, its later amendments, and the principles of good clinical practice. The study protocol was approved by the Ethics Committee of East Slovakia Oncological Institute on 27 May 2021 (approval code, EK/2/05/2021). The committee waived the need for the patients’ informed consent due to the retrospective nature of the data collection.

### Statistical Analyses

Continuous variables are described by medians and interquartile ranges (IQRs). Categorical variables are described by absolute counts and percentages. The Shapiro–Wilk test was used to evaluate the normality of distribution of continuous variables. Mann–Whitney tests were used to compare differences in continuous variables (because many of them were not normally distributed) and *χ*^2^ tests/Fisher’s exact tests were used to compare differences in categorical variables. We also performed survival analyses by estimating the survival distributions using the Kaplan–Meier method and evaluated the significance using the log-rank test. The cut-off levels of continuous variables were chosen to be clinically meaningful. We also performed secondary analysis using multiple Cox proportional hazards regression modeling. In the secondary analysis, we used only predictors that were significant in the primary analysis and, similar to Bruix et al., adjusted them for BCLC [24]. The results are presented as the hazard ratio, its 95% confidence interval, and the *p*-value of the Wald’s test. All tests were performed at a 0.05 significance level. Data were analyzed using RStudio (version 1.2.1335; RStudio Team; Boston, MA, USA).

## 3. Results

A total of 368 patients were included in the final analysis. Fifty-four patients had NAFLD-related HCC and 314 patients had other etiology-related HCC (AFLD, HBV, or HCV). The patients’ demographics and baseline clinical characteristics are presented in Table 1. NAFLD-related HCC patients were younger (*p* = 0.0007), with a higher percentage of women (*p* < 0.001) compared to patients with other etiology-related HCC. There was also a lower proportion of patients who had liver cirrhosis at the time of HCC diagnosis in the NAFLD-related HCC subgroup (*p* < 0.0001). Forty-six patients (85%) had “HCC in liver cirrhosis due to NAFLD,” and only a small group of these patients (15%) had HCC in a non-cirrhotic liver. Understandably, patients from the NAFLD-related HCC subgroup had a higher prevalence of diabetes and metabolic syndrome when compared to other etiology-related HCC (*p* < 0.0001), and they also had lower total bilirubin (*p* = 0.005), a higher platelet count (*p* < 0.0001), higher PLR (*p* = 0.001), and lower APRI (*p* < 0.0001). Interestingly, patients with NAFLD-related HCC were diagnosed in more advanced BCLC stages when compared to patients with HCV-related HCC only (*p* = 0.02).

A short treatment summary stratified by BCLC stages is presented in Table 2. The most frequent treatments employed were tumor resection (BCLC 0–A), transarterial chemoembolization (BCLC B), and sorafenib (BCLC C), and patients from BCLC D were indicated for best supportive care only.

NAFLD-related HCC patients had metformin and statins in their chronic medication more frequently (*p* = 0.0008 and *p* = 0.001, respectively) (Table 3).

**Figure 2:**
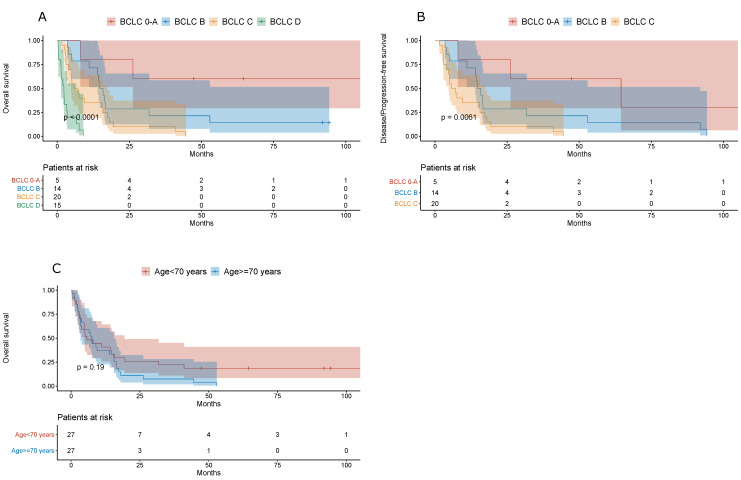
(**A**) Overall survival of NAFLD-related HCC patients. (**B**) Disease-/progression-free survival of NAFLD-related HCC patients. (**C**) Overall survival of NAFLD-related HCC patients stratified by age (cut-off, 70 years).

We confirmed a good prognostic performance of the BCLC staging system in predicting both the overall and disease-/progression-free survival of NAFLD-related HCC patients (*p* < 0.0001 and *p* = 0.0061, respectively). The overall survival was similar in NAFLD-related HCC patients younger and older than 70 years (Figure 2).

**Figure 3:**
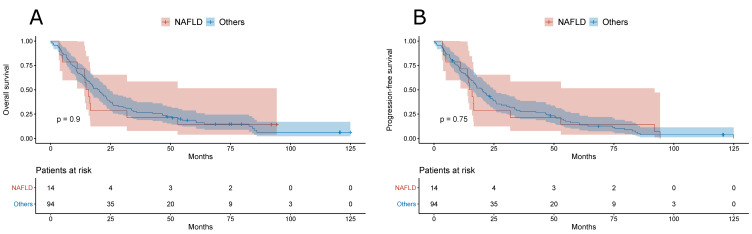
(**A**) Overall survival of patients with NAFLD- and other etiology-related BCLC B HCC. (**B**) Progression-free survival of patients with NAFLD- and other etiology-related BCLC B HCC.

**Figure 4:**
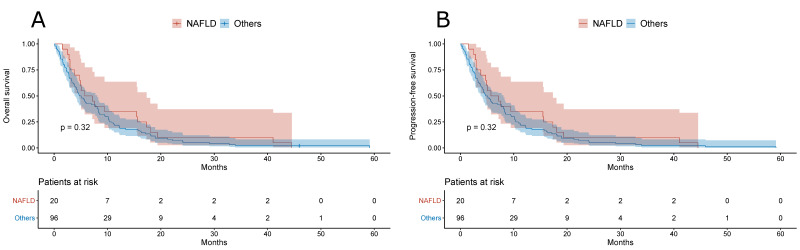
(**A**) Overall survival of patients with NAFLD- and other etiology-related BCLC C HCC. (**B**) Progression-free survival of patients with NAFLD- and other etiology-related BCLC C HCC.

Furthermore, there was no difference in the overall survival or in the progression-free survival between NAFLD- and other etiology-related HCC patients with BCLC B (Figure 3) or BCLC C (Figure 4).

**Figure 5:**
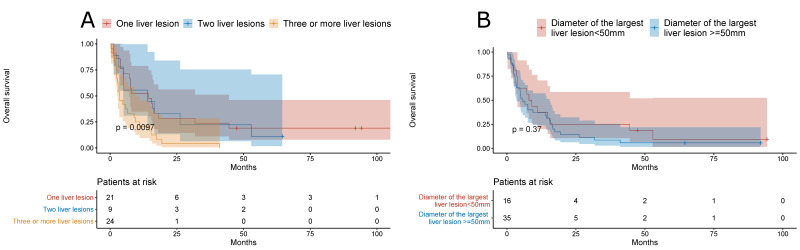
(**A**) Overall survival of NAFLD-related HCC patients stratified by the number of liver lesions. (**B**) Overall survival of NAFLD-related HCC patients stratified by the diameter of the largest lesion (cut-off value, 50 mm).

NAFLD-related HCC patients with three or more liver lesions had a shorter overall survival when compared to patients with one or two liver lesions only (*p* = 0.0097); however, the overall survival was similar in patients stratified by the diameter of the largest lesion (cut-off, 50 mm) (Figure 5).

**Figure 6:**
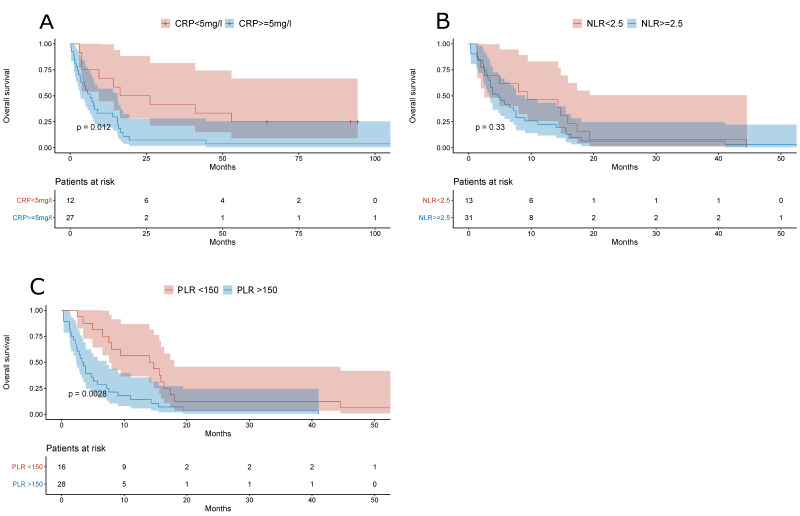
(**A**) Overall survival of NAFLD-related HCC patients stratified by the baseline CRP value (cut-off, 5 mg/L). (**B**) Overall survival of NAFLD-related HCC patients stratified by the baseline NLR (cut-off, 2.5). (**C**) Overall survival of NAFLD-related HCC patients stratified by the baseline PLR (cut-off, 150).

NAFLD-related HCC patients with baseline CRP values of ≥5 mg/L or with PLR ≥ 150 lived significantly shorter (*p* = 0.012 and *p* = 0.0028, respectively); on the contrary, there was no difference in the overall survival when an NLR cut-off value of 2.5 was used (Figure 6).

**Figure 7:**
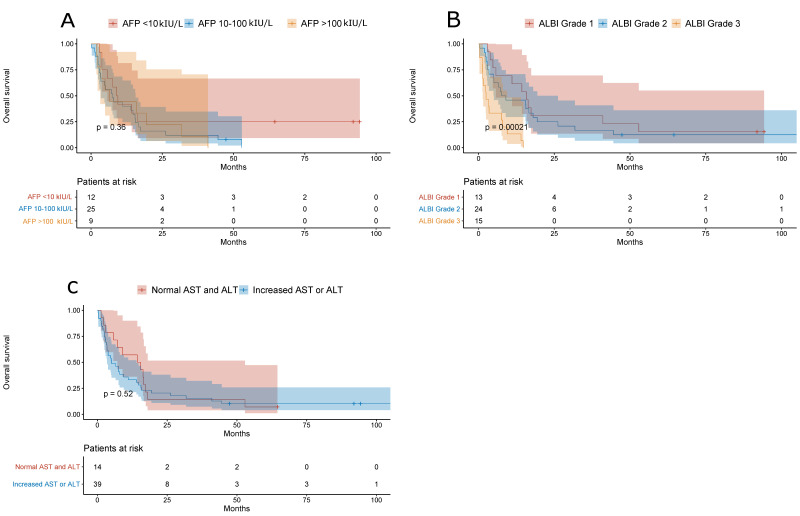
(**A**) Overall survival of NAFLD-related HCC patients stratified by the baseline AFP value (cut-off, 10 and 100 kIU/L). (**B**) Overall survival of NAFLD-related HCC patients stratified by ALBI grade. (**C**) Overall survival of NAFLD-related HCC patients with normal and increased serum aminotransferases.

NAFLD-related HCC patients with ALBI Grade 3 had worse overall survival when compared to patients with ALBI Grade 1 or 2 (*p* = 0.00021), and there was no difference in the overall survival between patients with normal or increased baseline serum aminotransferases and among patients stratified by baseline AFP values (cut-off, 10 and 100 µg/L) (Figure 7).

In the secondary analysis, PLR ≥ 150 and ALBI Grade 3 were associated with worse overall survival of NAFLD-related HCC patients after adjusting for the BCLC stages (HR = 2.17, 95% CI = 1.04–4.54 and HR = 3.20, 95% CI = 1.18–8.72, respectively) (Table 4).

## 4. Discussion

The pathogenesis of HCC in liver cirrhosis due to NAFLD is multifactorial. Factors associated with obesity, T2DM, or insulin resistance play a major role in the pathogenesis. There is an overproduction of leptin in adipose tissue, which has pro-inflammatory, proangiogenic, and profibrogenic roles, activates the Janus kinase pathway, and triggers carcinogenesis. In patients with NAFLD, there is an alteration of the bowel microbiota and the release of increased levels of lipopolysaccharide (LPS) from Gram-negative bacteria into the circulation, where LPS has a pro-inflammatory and carcinogenic effect. Liver fat accumulation leads to the generation of reactive oxygen species and the overproduction of saturated and monounsaturated free fatty acids, with the subsequent triggering of cellular signaling cascades and activation of pro-carcinogenic pathways. Furthermore, overproduction of insulin and insulin-like growth factor 1 leads to the activation of multiple oncogenic pathways. Increased iron absorption in patients with nonalcoholic steatohepatitis also triggers carcinogenic mechanisms. Genetics plays a principal role in the development of NAFLD-related HCC. NAFLD patients with PNPLA3 rs738409 c444C > G minor allele (encoding the I148M variant) are known to be at high risk of HCC. However, HCC can also arise in non-cirrhotic NAFLD, even in patients without fibrosis or with minimal fibrosis, where the malignant transformation of an obesity-associated inflammatory hepatocellular adenoma is possible [25,26].

Of the cohort of patients with an identified cause of underlying liver disease, 12.59% of patients had NAFLD-related HCC. Furthermore, more than 9% of patients with HCC had cryptogenic liver cirrhosis. NAFLD is believed to be the most common cause of cryptogenic liver cirrhosis [27]; therefore, up to almost 22% of patients had HCC with an underlying diagnosis of NAFLD. Our data correlate with studies conducted in France and the United Kingdom, where in a cohort of patients with HCC diagnosed after 2010, NAFLD was the underlying diagnosis in 19.5% and 34.8%, respectively. In both studies, the percentage of NAFLD in HCC patients increased over time. In the U.K. study, less than 10% of HCC patients diagnosed before 2010 had NAFLD, but among patients diagnosed with HCC after 2010 NAFLD was an underlying hepatologic diagnosis in 34.8% [28,29]. We can conclude that the proportion of NAFLD among HCC patients is increasing, and NAFLD is likely to become the leading cause of HCC in developed countries in the near future.

In our analysis, we included only patients with HCC based on verified NAFLD. Fifteen percent of them did not have liver cirrhosis at the time of diagnosis, which is the lower limit of data reported in the literature [4]. According to some reports, a minority of HCC in non-cirrhotic livers may arise from the transformation of a hepatic adenoma [30]. Patients with NAFLD and HCC were significantly more likely to have a tumor in the non-cirrhotic liver compared to patients with HCC related to other underlying liver diseases, which is consistent with the literature data [4]. Survival of patients with NAFLD-related HCC in non-cirrhotic liver is comparable to patients with NAFLD-related HCC and liver cirrhosis [31]. We did not compare survival between the two patient groups in our cohort due to the small number of patients.

Females accounted for more than 60% of patients with NAFLD-related HCC, which is in contrast to the literature data, where the majority of patients with NAFLD-related HCC are men [32]. This contradiction may be explained by three facts. The majority of female patients with NAFLD-related HCC were postmenopausal, where the protective effect of hormones in the pathophysiology of HCC development is much lower. The second fact is that obesity is present in approximately one-third of Slovak women and only one-quarter of Slovak men [33]. The third fact is that alcohol consumption in Slovakia is extremely high [34]. The genetic predisposition for NAFLD overlaps with the genetic predisposition for alcoholic liver disease [35]; many patients evaluated as having alcoholic liver disease in fact have an overlap of NAFLD and alcoholic liver disease. The most common underlying liver disease in our cohort of HCC patients was alcoholic liver cirrhosis. Patients with alcoholic liver cirrhosis accounted for over 50% of the HCC cases, and the proportion of males in this subgroup was very high (almost 75%). A portion of these patients are likely to have an overlap of NAFLD and alcoholic liver disease. Patients with NAFLD were diagnosed with HCC at an older age compared to patients with other etiology-related HCC. The mean age at diagnosis was approximately 70 years, which is consistent with the literature data [36]. However, older patients with NAFLD-related HCC had comparable survival to younger patients with HCC related to other liver diseases.

Patients with NAFLD were diagnosed with HCC at more advanced stages of the disease. Almost two-thirds of patients were diagnosed at stage BCLC C or D, and over a quarter of patients at stage D where no treatment is available. These data are due to a lack of HCC surveillance in NAFLD patients [2]. NAFLD-related HCC patients in our cohort had a statistically more advanced disease stage at diagnosis compared to patients with HCC related to chronic hepatitis C. Despite the fact that the first-line treatment of patients with NAFLD-related HCC was almost always consistent with the current guidelines for HCC management, the prognosis of patients in this group was not good because of the advanced disease at diagnosis, especially in patients with BCLC stage C or D. The importance of adequate HCC surveillance was demonstrated in a Brazilian study, where more than half of patients with NAFLD or cryptogenic liver cirrhosis had HCC detected at BCLC stage 0 or A, almost one-quarter at stage B, and only 12% at stage D. One-year overall survival in this study was 81% and two-year overall survival was 66% [37]. The overall survival of NAFLD-related HCC patients in our cohort in both BCLC B and BCLC C was comparable to the overall survival of patients with HCC related to other liver diseases.

We did not compare overall survival between NAFLD-related HCC and other etiology-related HCC patients with BCLC A. This was because we were worried about the small sample size in NAFLD-related patients (*n* = 5), which could have resulted in the low power of the test. Furthermore, we did not compare overall survival between patients in BCLC D, because these patients did not receive any specific treatment and were indicated for the best supportive care only. As mentioned above, the overall survival of patients with NAFLD-related HCC in BCLC stages B and C was comparable to that of patients with other etiology-related HCC in the same BCLC stages. This suggests that the overall survival is dependent on the general condition of patients and not on the etiology of HCC itself. Although many patients with viral hepatitis-related HCC received specific treatment, and even though many patients with alcoholic liver disease-related HCC stopped drinking alcohol, patients from these subgroups had similar overall survival to patients with NAFLD-related HCC from the same BCLC stage, whom we did not treat with any specific treatment. This observation suggests that the general condition and the BCLC staging have significant prognostic potential and not the etiology of HCC itself.

NAFLD HCC patients were more likely to have metabolic syndrome and type 2 diabetes mellitus and were more likely to take metformin and statin than patients with HCC related to other liver diseases. However, only one-sixth of NAFLD HCC patients took statin and just under one-quarter took metformin. Both of these drugs can have a preventive effect for HCC development [38,39]; therefore, the absence of these drugs in the majority of patients may have contributed to the development of HCC.

In our study, we also evaluated predictive factors of the overall survival in NAFLD-related HCC patients. Patients with multifocal HCC had a worse prognosis, whereas the size of the lesion did not prove to be a predictive factor, probably due to the fact that patients with a small diameter of the largest lesion often had multifocal HCC. The AFP value did not predict overall survival either. Prognostic factors assessing liver functional capacity or the intensity of subclinical inflammation may also predict the prognosis of HCC patients [40,41,42,43,44]. In our cohort of patients, ALBI grade predicted overall survival. Patients with C-reactive protein at diagnosis <5 mg/L and a platelet-to-lymphocyte ratio <150 had a better prognosis, but the neutrophil-to-lymphocyte ratio had no prognostic significance in this group of patients. In secondary analysis, we confirmed that both PLR and ALBI are predictors of overall survival, even after adjusting for BCLC.

As mentioned above, patients with NAFLD and higher stage fibrosis or even liver cirrhosis have a higher risk of developing HCC. Prior to the REGENERATE study, no drug with an antifibrotic effect in NASH was available [45,46]. The introduction of obeticholic acid into the routine treatment of NASH patients is limited by the high cost of the treatment. Standard anticancer therapy for HCC has equal efficacy in patients with NAFLD compared to HCC related to other liver diseases. However, patients with NAFLD-related HCC respond worse to experimental immunotherapy for HCC. An analysis of three phase III clinical trials showed that patients with HCC and NASH had a worse response to treatment with inhibitors of programmed-death ligand 1 compared to patients with HCC related to other liver diseases [47].

Our study was the first to monitor the clinical course and prognostic factors of patients with NAFLD-related HCC. However, this study has several limitations: The retrospective design and a large heterogeneity in the cohort, as well as the small number of patients, especially in the BCLC stages 0–A, and the small number of HCC patients in the non-cirrhotic stage, which limited the quality of the study.

NAFLD is a common cause of HCC. The prevalence of HCC in the European population is on an upward trend, and an increase in the prevalence of NAFLD HCC from 93% to 125% can be expected between 2016 and 2030 [48]. NAFLD is likely to soon become the most common etiological cause of HCC in the civilized world. Lifestyle changes and the development of new drugs for NASH may contribute to the reduction in HCC incidence in NAFLD in the future. Improved surveillance may lead to the HCC detection at early stages of the disease, when curative treatment is possible, while the development of new anticancer therapeutic strategies may contribute to prolonged overall survival.

## 5. Conclusions

Approximately one-eighth of HCC patients had NAFLD-related HCC. Patients with NAFLD-related HCC were older than patients with other etiologies-related HCC, and they were diagnosed in a more advanced stage of the disease. We did not find any difference in the overall survival or in the progression-free survival between NAFLD- and other etiology-related HCC patients in BCLC stages B or C. We confirmed the good prognostic performance of the BCLC staging system in predicting both the overall and disease-/progression-free survival of NAFLD-related HCC patients. Furthermore, both PLR and ALBI are significant predictors of the overall survival independent of the BCLC staging system.

## Figures and Tables

**Table 1:** Clinical and demographic characteristics of patients with HCC.

		Other Etiology-Related HCC	NAFLD-Related HCC	*p*
Patients		314 (85.33%)	54 (14.67%)	
Male patients		224 (71.34%)	21 (38.89%)	<0.001
Female patients		90 (28.66%)	33 (61.11%)	0.0007
Age at diagnosis	Years	65 (60–71)	69.5 (64.2–75.8)	
BCLC				
0–A		57 (18.15%)	5 (9.26%)	
B		94 (29.94%)	14 (25.93%)	
C		96 (30.57%)	20 (37.04%)	
D		67 (21.34%)	15 (27.78%)	0.27
Cirrhosis at diagnosis		311 (99.04%)	46 (85.19)	<0.0001
Diabetes		99 (31.53%)	41 (75.93%)	<0.0001
Child-Pugh score		6 (5–8)	7 (5–8)	0.14
Performance status		1 (1–1)	1 (1–2)	0.19
Metabolic syndrome		70 (22.29%)	36 (66.67%)	<0.0001
Total bilirubin	µmol/L	24.1 (15.5–40.4)	18.9 (11.3–27.6)	0.005
Direct bilirubin	µmol/L	10.6 (6.1–21.6)	8.85 (5.05–17.9)	0.11
AST	µkat/L	1.10 (0.69–1.94)	0.91 (0.55–1.56)	0.08
ALT	µkat/L	0.69 (0.44–1.28)	0.69 (0.42–1.19)	0.41
GMT	µkat/L	2.55 (1.23–4.64)	2.44 (1.28–5.85)	0.38
ALP	µkat/L	2.34 (1.68–3.58)	2.26 (1.89–4.03)	0.36
Albumin	g/L	33.2 (28–38)	35 (28.7–39.3)	0.34
CRP	mg/L	12.6 (3.43–35.0)	9.8 (4.21–45.6)	0.63
Total cholesterol	mmol/L	4.32 (3.37–4.99)	4.12 (3.69–5.07)	0.64
HDL-C	mmol/L	0.92 (0.69–1.37)	1.05 (0.81–1.11)	0.90
LDL-C	mmol/L	2.61 (1.82–3.2)	2.16 (1.76–2.92)	0.56
Triglycerides	mmol/L	1.13 (0.74–1.5)	1.32 (1.0–1.65)	0.12
Na	mmol/L	139 (134–140)	138 (137–142)	0.01
Urea	mmol/L	5.3 (4.2–7.7)	5.4 (4.1–6.5)	0.38
Creatinine	µmol/L	81.3 (69.2–103)	81 (70.8–99)	0.81
Neutrophils	×10^9^	3.85 (2.62–5.59)	4.58 (3.06–7.34)	0.04
Lymphocytes	×10^9^	1.09 (0.71–1.72)	1.35 (1.03–1.61)	0.11
Platelets	×10^9^	137 (93–208)	223 (129–317)	<0.0001
NLR		3.65 (2.23–5.38)	3.68 (2.35–6.83)	0.58
PLR		125 (82.2–202)	177 (122–247)	0.001
APRI		0.897 (0.48–1.73)	0.489 (0.25–0.89)	<0.0001
APRI ≥1.0		146 (46.50%)	11 (20.37%)	0.0007
DFS/PFS	Months	5.22 (1.97–16.7)	4.98 (2.78–9.34)	0.76
OS	Months	8.38 (2.3–24.4)	7.37 (2.97–16.5)	0.68

The category “other etiology-related HCC” is composed of HBV-, HCV-, and alcohol fatty liver disease-related HCC. Continuous variables are presented as medians and interquartile ranges; categorical variables are presented as counts and percentages. ALT, alanine aminotransferase; ALP, alkaline phosphatase; AST, aspartate aminotransferase; BCLC, Barcelona Clinic Liver Cancer; CRP, c-reactive protein; DFS, disease-free survival; GMT, gamma-glutamyl transferase; HCC, hepatocellular carcinoma; HDL, high-density lipoprotein; Na, natrium; OS, overall survival; PFS, progression-free survival.

**Table 2:** Treatment of patients with hepatocellular carcinoma.

	Other Etiology-Related HCC (*n* = 314)	NAFLD-Related HCC (*n* = 54)
BCLC 0–A		
Tumor resection	15 (4.78%)	5 (9.26%)
Radiofrequency ablation	13 (4.14%)	0
Transarterial chemoembolization	9 (2.87%)	0
Best supportive care	7 (2.23%)	0
Liver transplantation	13 (4.14%)	0
BCLC B		
Tumor resection	10 (3.18%)	
Radiofrequency ablation	6 (1.91%)	1 (1.85%)
Transarterial chemoembolization	56 (17.83%)	0
Sorafenib	9 (2.87%)	10 (18.52%)
Best supportive care	5 (1.59%)	1 (1.85%)
Chemotherapy	1 (0.32%)	2 (3.70%)
Liver transplantation	7 (2.23%)	0
BCLC C		
Tumor resection	1 (0.32%)	
Transarterial chemoembolization	8 (2.55%)	0
Sorafenib	60 (19.11%)	0
Best supportive care	24 (7.64%)	17 (31.48%)
Sorafenib + transarterial chemoembolization	1 (0.32%)	3 (5.56%)
Chemotherapy	2 (0.64%)	0
BCLC D		
Sorafenib	0	0
Best supportive care	67 (21.34%)	15 (27.78%)

The category “other etiology-related HCC” is composed of HBV-, HCV-, and alcohol fatty liver disease-related HCC. BCLC, Barcelona Clinic Liver Cancer.

**Table 3:** Chronic medication of patients with HCC unrelated to oncologic treatment.

	Other Etiology-Related HCC (*n* = 314)	NAFLD-Related HCC (*n* = 54)	*p*
Statins	12 (3.82%)	9 (16.67%)	0.001
Low molecular weight heparin	14 (4.46%)	4 (7.41%)	0.32
Metformin	24 (7.64%)	13 (24.07%)	0.0008
Ursodeoxycholic acid	76 (24.20%)	8 (14.81%)	0.16

The category “other etiology-related HCC” is composed of HBV-, HCV-, and alcohol fatty liver disease-related HCC.

**Table 4:** Factors associated with overall survival in patients with NAFLD-related HCC (after adjusting for BCLC stage).

	Hazard Ratio (95% Confidence Interval)	*p*-Value
Number of liver lesions		
1	Ref.	
2	1.58 (0.6515–3.8400)	0.31
≥3	1.38 (0.6976–2.7390)	0.35
CRP		
<5 mg/L	Ref.	
≥5 mg/L	1.41 (0.5366–3.706)	0.49
PLR		
<150	Ref.	
≥150	2.17 (1.0408–4.5440)	0.04
ALBI		
Grade 1	Ref.	
Grade 2	1.37 (0.5899–3.1830)	0.46
Grade 3	3.20 (1.1771–8.7190)	0.02

ALBI, albumin-bilirubin Grade; BCLC, Barcelona Clinic Liver Cancer; CRP, C reactive protein; HCC, hepatocellular carcinoma; NAFLD, nonalcoholic fatty liver disease; PLR, platelet-to-lymphocyte ratio.

## Data Availability

The data used to support the findings of this study are available from the corresponding author upon request.
